# Mast cells and hypoxia drive tissue metaplasia and heterotopic ossification in idiopathic arthrofibrosis after total knee arthroplasty

**DOI:** 10.1186/1755-1536-3-17

**Published:** 2010-09-01

**Authors:** Theresa A Freeman, Javad Parvizi, Craig J Dela Valle, Marla J Steinbeck

**Affiliations:** 1 Department of Orthopaedic Surgery, Thomas Jefferson University, 1015 Walnut Street, Suite 501, Philadelphia, PA 19107, USA; 2 The Rothman Institute of Orthopedics at Thomas Jefferson University, 925 Chestnut Street, Philadelphia, PA 19107, USA; 3 Department of Orthopaedic Surgery, Rush University Medical Center, 1725 W. Harrison Street, Suite 1063, Chicago, IL 60612, USA; 4 School of Biomedical Engineering and College of Drexel Medicine, Drexel University, 3120 Market Street, 323 Bossone, Philadelphia, PA 19104, USA

## Abstract

**Background:**

Idiopathic arthrofibrosis occurs in 3-4% of patients who undergo total knee arthroplasty (TKA). However, little is known about the cellular or molecular changes involved in the onset or progression of this condition. To classify the histomorphologic changes and evaluate potential contributing factors, periarticular tissues from the knees of patients with arthrofibrosis were analyzed for fibroblast and mast cell proliferation, heterotopic ossification, cellular apoptosis, hypoxia and oxidative stress.

**Results:**

The arthrofibrotic tissue was composed of dense fibroblastic regions, with limited vascularity along the outer edges. Within the fibrotic regions, elevated numbers of chymase/fibroblast growth factor (FGF)-expressing mast cells were observed. In addition, this region contained fibrocartilage and associated heterotopic ossification, which quantitatively correlated with decreased range of motion (stiffness). Fibrotic, fibrocartilage and ossified regions contained few terminal dUTP nick end labeling (TUNEL)-positive or apoptotic cells, despite positive immunostaining for lactate dehydrogenase (LDH)5, a marker of hypoxia, and nitrotyrosine, a marker for protein nitrosylation. LDH5 and nitrotyrosine were found in the same tissue areas, indicating that hypoxic areas within the tissue were associated with increased production of reactive oxygen and nitrogen species.

**Conclusions:**

Taken together, we suggest that hypoxia-associated oxidative stress initiates mast cell proliferation and FGF secretion, spurring fibroblast proliferation and tissue fibrosis. Fibroblasts within this hypoxic environment undergo metaplastic transformation to fibrocartilage, followed by heterotopic ossification, resulting in increased joint stiffness. Thus, hypoxia and associated oxidative stress are potential therapeutic targets for fibrosis and metaplastic progression of idiopathic arthrofibrosis after TKA.

## Background

Limited range of motion is a disabling complication of total knee arthroplasty (TKA) [1-8]. Idiopathic arthrofibrosis, clinically defined as abnormal tissue scarring within the joint, represents the most severe form of stiffness. Even with surgical intervention or revision arthroplasty, the formation of dense fibrous tissue and tissue contractions can recur, leading to increased severity of the fibrotic condition and eventual disability, with all the negative psychological and societal implications this carries [8]. Unfortunately, there is no clear method by which patients who are at risk of developing arthrofibrosis can be identified.

It is known that arthrofibrosis develops in response to surgical intervention in approximately 3-4% of patients undergoing TKA, and the resulting pain and loss of range of motion leads to patient disability. Thus, to have the possibility of developing a diagnostic test to prescreen patients undergoing TKA and/or of implementing postoperative treatments that may improve the functional outcome, it is important to understand the pathoetiology behind the condition. In this study, we aimed to identify the specific factor(s) responsible for initiating tissue fibrosis, metaplasia and other histological changes that occur in arthrofibrosis.

In agreement with other investigators, we have previously reported the presence of aggressive fibroblast proliferation and heterotopic ossification (HO) in arthrofibrotic tissues [6,8-13]. In general, fibrogenesis resulting from tissue injury is characterized by fibroblast proliferation, excessive synthesis and accumulation of extracellular matrix (ECM) components, and reduced ECM remodeling. The accumulation of unremodeled ECM can result in impaired blood flow and oxygen delivery to the tissue, which leads to tissue hypoxia [14-16]. In response to hypoxia, expression of glycolytic enzymes such as lactate dehydrogenase (LDH)5, are induced to promote glycolysis as a source of ATP [17-20]. In turn, tissue hypoxia stimulates the recruitment of peripheral blood fibrocytes to the wound site, where microenvironmental factors have been shown to induce their 'transdifferentiation' into other cell types [21-24]. *In vitro*, dermal fibroblasts have been shown to transdifferentiate into chondrocytes in response to a number of local factors [25,26], such as mechanical stress, growth factors (for example, transforming growth factor (TGF)-β_1_, fibroblast growth factor (FGF)) and hypoxia [17,24,27,28]. All three factors induce proteoglycan synthesis, which is an essential component for attachment and transdifferentiation of fibroblasts into chondrocytes [29,30].

Reactive oxygen and nitrogen species (RONS) production in association with hypoxic conditions has been previously reported [31], and we have reported the involvement of inflammation and RONS in the development of this condition [13]. The production of RONS has been shown to stimulate degranulation of mast cells, another cell type involved directly in the development of other fibrogenic diseases. In fibroproliferative disorders of the skin and gastrointestinal tract, mast cells undergo proliferation and activation, and show increased expression of mast cell-specific chymase [32-35]. Chymase-expressing mast cells are generally classified as non-immune mast cells, differentiating them from mast cells that only express tryptase and that are involved in allergic and parasitic diseases [34]. The release of chymase results in the cleavage and activation of TGF-β_1 _[33,35], which can stimulate fibroblast proliferation, ECM production and transdifferentiation of fibrotic tissue to fibrocartilage [36,37]. The presence of increased mast cell numbers has also been observed after tissue injury in patients with fibrodysplasia ossificans progressiva, a disease marked by heterotopic ossification [38]. Thus, the potential for an inter-related involvement of hypoxia, RONS and mast cells in the development of arthrofibrosis and tissue metaplasia exists.

The current study was undertaken to evaluate and classify the histomorphologic pathology that characterizes arthrofibrosis, with the expectation that the results would promote an understanding of the possible mechanisms behind the fibrosis and metaplastic changes that occur during disease progression.

## Results

### Patient cohort information

Tissues were taken from 10 patients diagnosed with idiopathic arthrofibrosis. Patient information including age, sex, body mass index (BMI), years since initial surgery, pre-existent comorbidities, and functional scores, particularly range of motion (ROM) were obtained from clinical charts (Table 1). The control group comprised 10 patients matched for age and BMI (4 men (mean ± SD age 64 ± 1.0 years, BMI 28.4 ± 2.6) and 6 women (age 61 ± 7.0 years, BMI 38.7 ± 0.25)) who were undergoing primary TKA for treatment of osteoarthritis (OA) (Table 1).

### Evaluating the presence of bone within the arthrofibrotic tissue

**Figure 1:**
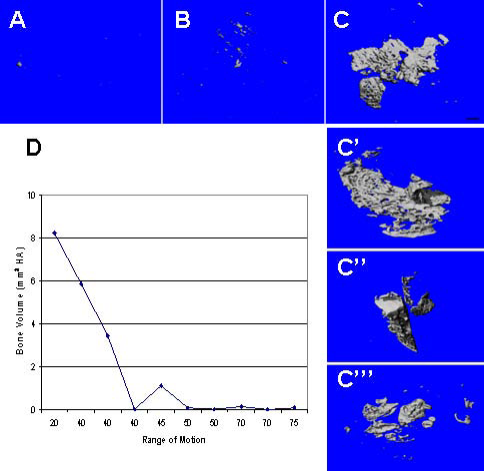
**Microcomputed tomography (microCT) analysis of the periarticular tissue and comparison of bone volume (BV) and range of motion (ROM)**. Representative microCT images are shown for **(A) **minimal calcification in pre-surgical osteoarthritic tissues (n = 5), **(B) **low BV group (0.07 ± 0.02 mm^3 ^of hydroxyapatite; n = 5) and **(C) **high BV group (7.4 ± 2.7 mm^3^; n = 5). Calcified regions adjoining the fibrocartilage were confirmed by microCT analysis to have the appearance and density consistent with newly formed bone. (**D**) ROM and BV comparisons; the closer the patient ROM was to normal flexion, the lower the amount of tissue calcification or BV. Once the ROM was restricted to < 50 degrees, BV increased, and reached a peak when ROM was ≤ 20 degrees.

Microcomputed tomography (microCT) and 3 D reconstructions of the tissue scans were used to evaluate the presence of bone within the arthrofibrotic tissue, and indicated that all 10 patient tissues contained mineralized material with a density consistent with that of bone. Five of the 10 patient samples had extensive amounts of bone as measured by bone volume (BV). Three representative images are shown in Figure 1 for the OA, low BV and high BV arthrofibrotic groups. Some bone was detected in the OA tissues, but it was significantly (*P *< 0.05) less than that observed in the low BV group (Figure 1A; n = 10). The low BV group showed increased but diffuse mineralization (Figure 1B; n = 5), and the high BV group contained large bone-like mineralized deposits (Figure 1C to 1C'; n = 5). However, blindly evaluated radiographs for all patients showed no evidence of heterotopic ossification (HO), indicating that the bone identified by microCT was below the resolution of plain radiography.

To evaluate correlations between BV and patient clinical information, we compared patient variables and tissue BV. The only significant correlation was between ROM and BV; the closer the ROM was to normal flexion, the lower the amount of BV (Figure 1D). Once the ROM was restricted to < 50 degrees, BV increased, and reached a peak at a ROM of ≤ 20 degrees. For subsequent analyses, patients were grouped on the basis of the amount of BV in their tissues.

### Morphological and cell survival aspects of arthrofibrotic tissue

**Figure 2:**
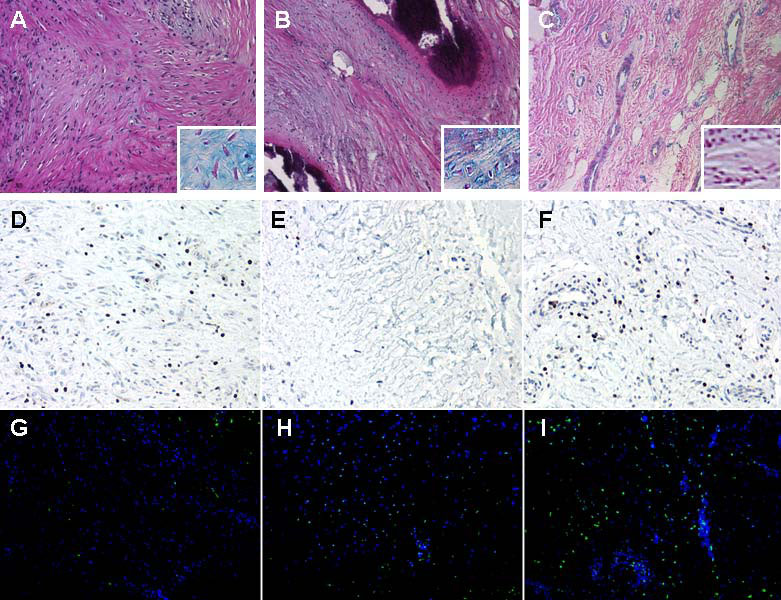
**Tissue morphology, cell survival and programmed cell death in periarticular tissue**. Representative images are shown for **(A, D, G) **fibrotic **(B, E, H)**, fibrocartilage with calcification **(C, F, I) **and vascular regions. Tissues were stained **(A-C)**with hematoxylin and eosin and alcian blue (insets enlarged 100%**)**, **(D-F) **Bcl-2 and terminal dUTP nick end labeling (TUNEL); **(G-I) **green apoptotic cells and 4',6-diamidino-2-phenylindole(DAPI)-stained blue stained nuclei. Scale bar = 100 μm.

To characterize tissue removed at the time of the revision surgery, tissue sections were prepared and stained. Systematic analysis of the tissues and stains by brightfield microscopy revealed three distinct morphological regions within each sample. These regions (representative images shown in Figure 2) were classified as fibrotic (Figure 2A, D, G), fibrocartilage with calcification (Figure 2B, E, H) and vascular (Figure 2C, F, I). The morphologic features were highlighted by staining with hematoxylin and eosin to show the distinct characteristics of each region (Figure 2A-C). To evaluate the proteoglycan content, tissues were stained with alcian blue. There was increased proteoglycan deposition within the fibrotic areas and adjacent fibrocartilage regions (Figure 2A-C, insets). By contrast, very little proteoglycan deposition was observed in the vascular regions.

The fibrotic regions (Figure 2A) contained the highest cell density per cubic millimeter compared with the fibrocartilage with calcification (Figure 2B) and the vascular regions (Figure 2C). Cells within this portion of the tissue showed the characteristic spindle-shaped morphology. Other distinctive features were the disorganized nature of the collagen fibers and decreased vascularity. Adjacent to the fibrotic areas were regions showing characteristic features of fibrocartilage with calcification (Figure 2B). The cells in this region had a distinctly rounded shape surrounded by a clear lacunar space, similar to mature chondrocytes, and lay adjacent to the solid dark region of mineralization (confirmed with alizarin red staining, data not shown). Finally, the outer edges of the arthrofibrotic tissue showed high vascularity with the presence of both large and small blood vessels (Figure 2C).

To determine whether increases in fibroblast number were related to decreased cell turnover, we assessed cell survival and apoptosis. Cell survival was evaluated by Bcl-2 immunohistochemistry, and apoptosis was determined by terminal dUTP nick end labeling (TUNEL) counterstained with 4',6-diamidino-2-phenylindole (DAPI), a nuclear-specific stain, indicating total cell number. Both the fibrotic and vascular regions showed increased Bcl-2 staining (Figure 2D-F). The highest number of apoptotic, TUNEL-positive (green), blue fluorescent nuclei was observed in the vascular region (Figure 2I), with a few apoptotic cells in the fibrocartilagenous regions (Figure 2H) and almost none in the fibrotic region (Figure 2G).

### Involvement of hypoxia in tissue metaplasia

**Figure 3:**
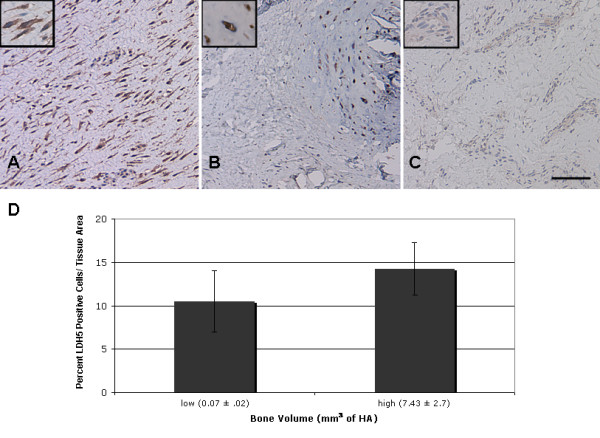
**Immunohistochemical stain of arthrofibrotic tissue for lactate dehydrogenase (LDH)5**. Representative LDH5 immunohistochemical results are shown for **(A) **fibrotic regions, (**B**) fibrocartilage/calcified regions and (**C**) vascular regions of the tissue. **(D) **Image analysis of LDH5 for the low and high bone volume (BV) groups. There was no significant difference between the two groups. The high magnification insets emphasize the increased LDH5 expression in fibroblasts and chondrocytes. Scale bar = 250 μm.

Given the predominantly low vascularized nature of the fibrotic and fibrocartilage regions of the arthrofibrotic tissue and the lack of apoptotic cells, we evaluated the role of hypoxia and anaerobic glycolysis in cell survival. To evaluate the hypoxic status and glycolytic index of the arthrofibrotic tissue, we measured the levels of LDH5 by immunohistochemistry. We found that LDH5 was strongly expressed in the avascular periarticular fibrous and fibrocartilage tissue regions (Figure 3A, B), which is consistent with the metabolic status of cartilage. Area analysis of fibrotic regions indicated that LDH5 expression level was high, regardless of the amount of bone present (Figure 3D). No LDH5 was detected in the vascularized region (Figure 3C) or in the OA cohort tissues (data not shown).

### Involvement of RONS and mast cells in tissue fibrosis and metaplasia

**Figure 4:**
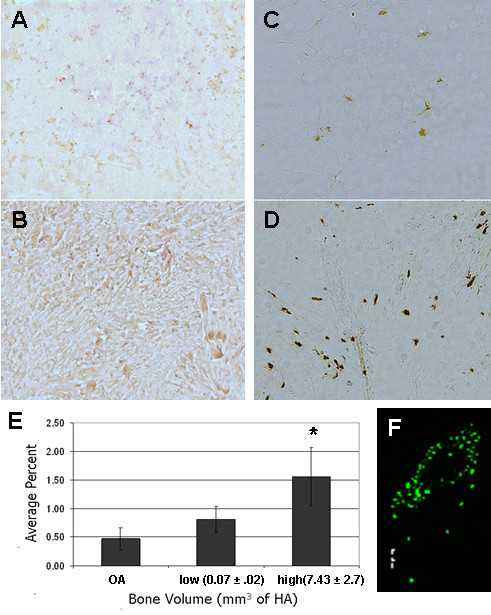
**Immunohistochemical stain of arthrofibrotic tissue for nitrotyrosine, chymase and fibroblast growth factor (FGF)**. Representative immunohistochemical results are shown for nitrotyrosine in **(A) **osteoarthritis (OA) tissue and (**B**) arthrofibrotic tissue, and chymase in (**C**) OA tissue and (**D**) arthrofibrotic tissue. **(E) **Image analysis of mast cell numbers for the low and high bone volume (BV) groups, compared with the OA cohort. There was a significant difference between all three groups (*P *< 0.05). (**F**) Representative immunohistochemical result of FGF expression in mast cells.

To assess hypoxia-associated RONS production and the potential colocalization of the observed protein nitrosylation with mast cells, tissues obtained during primary TKA surgery (OA tissue) and arthrofibrotic tissues were subjected to nitrotyrosine and chymase immunohistochemistry. Elevated levels of nitrosylated ECM proteins were observed in the arthrofibrotic tissues (Figure 4B) compared with the OA tissues (Figure 4A). In the same areas, increased numbers of chymase expressing mast cells were observed (Figure 4C, D). The chymase-positive mast cells also expressed FGF (Figure 4F), a factor involved in mediating fibroblast proliferation and chondrocyte differentiation [36,37]. Image analysis showed increased mast cell numbers in both the vascular and fibrotic regions, compared with OA tissues, whereas fibrocartilage regions had few or no mast cells (Figure 4E). The increase in mast cell number was directly related to the increase in BV. The number of mast cells in tissue from patients with high BV was almost double that of the low BV group.

## Discussion

**Figure 5:**
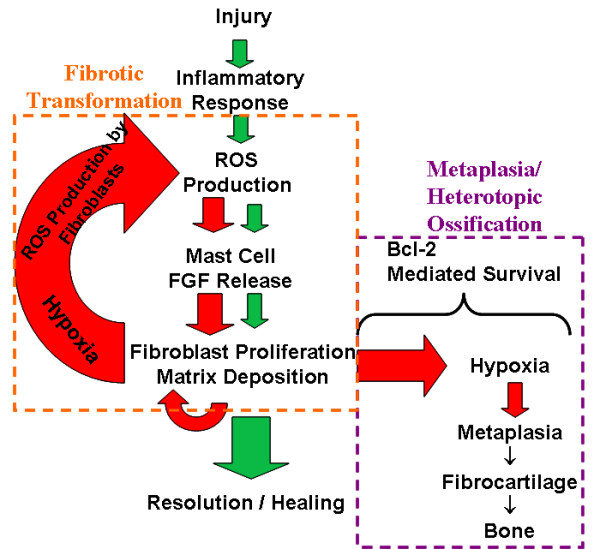
**Summary of findings of arthrofibrotic progression**. Green arrows indicate normal healing pathway, red arrows highlight disease progression pathway. The orange box indicates the etiology of the oxidative fibrotic transformation process supported by our previous studies [13]. The purple box indicates the pathology of metaplastic heterotopic ossification, supported by this study.

Our results show a direct correlation between the amount of bone formed during heterotopic ossification within the arthrofibrotic tissue and restricted ROM. Thus, the extent of the fibrogenic response and metaplastic changes directly relate to the degree of joint immobility and corresponding severity of arthrofibrosis. In addition, the arthrofibrotic tissue had regions that were fibrotic or contained fibrocartilage, both of which were predominantly avascular, with cells expressing LDH5, a hypoxia-specific gene. Despite the hypoxic environment, cellular apoptosis was not present. The fibrotic tissue also contained nitrosylated proteins concordant with elevated numbers of chymase and FGF producing mast cells, indicative of oxidative stress and increased fibroproliferative factors driving cell survival and proliferation. Based on these findings, we believe that hypoxia, hypoxia-associated oxidative stress, and mast cells drive the proliferation and survival of fibroblasts and their metaplastic conversion to fibrocartilage, which, through the process of endochondral ossification, results in heterotopic bone formation (Figure 5). These findings, together with the ultrastructure of the tissue divided into morphologically distinct regions of fibrosis, fibrocartilage and adjacent HO, represent a pathological timeline of the fibrogenic metaplastic process. Figure 5 is a schematic showing the normal progression of wound healing (green arrows) contrasted with the progression of abnormal healing, fibrosis and metaplastic changes (red arrows).

In a previous study, Ries *et al. *[6] reported histologic evidence of tissue fibrosis, and based on radiography, HO, in five of six arthrofibrotic knees evaluated. Retrospective studies by two other groups also demonstrated evidence of HO in arthrofibrotic knees, but the conclusions reached about the role of HO in stiffness differed between the two studies [9,10]. Furia *et al. *reported that 19 of 70 patients (27%) developed HO, and that 73% of patients with pre-existing heterotopic bone at other sites developed HO in the index knee. This group found a correlation between HO and a limitation of postoperative knee flexion. Furthermore, they suggested that preoperative measurement of spinal bone mineral density (BMD) might identify those with increased lumbar BMD, who are at risk of developing HO. In a separate study of 500 patients receiving cemented TKAs, the overall incidence of HO was reported to be 15% [10]. This group concluded that patients who developed HO tended to be heavier than average and that there was a male predilection, but concluded that HO did not appear to have a major influence on the outcome of TKA. The discrepancy of these findings may reflect the use of radiography to identify the presence of HO and the insensitivity of this approach to identify small amounts of bone formation, considering the HO observed in tissues by microCT and the lack of radiographic evidence of HO in the current study.

In the current study, we extended the original clinical study by Ries *et al. *[6], and found that the arthrofibrotic tissues consist of three distinct regions. Two of these regions were fibrotic, one was a highly vascularized area around the periphery of the excised tissue, and the other area contained disorganized matrix and increased proteoglycan content. The disorganization and increased proteoglycan content of the arthrofibrotic tissues is consistent with the ECM changes observed in other fibrotic tissues [39]. Altered synthesis of these macromolecules has serious implications for wound healing, inflammation and fibrosis, as they are involved in regulating numerous biologic processes [40]. Directly contiguous to the fibrotic areas was the third region type, consisting of avascular fibrocartilage, which undergoes endochondral ossification to form bone. Similar to the finding of Furia *et al*., the only significant correlation observed for the current cohort of patients was between decreased ROM and increased tissue BV [9].

The exact causes of fibroproliferative diseases (often defined as a wound-healing response that has gone unchecked, involving the lung, liver, kidney, skin and eye) is in many cases unknown [24,39,41]. One of the key events required for normal wound healing is apoptosis, and there is evidence for decreased pro-apoptotic pathway activation in fibroblasts taken from patients with pulmonary fibrosis [42]. We also found a low apoptotic index in the arthrofibrotic tissues in our study, suggesting that the loss of cell death may be a contributing factor in the fibrotic outcome for these patients.

Why arthrofibrosis or excessive scar formation develops in some patients and not others is at present unknown. It has been suggested that some patients may be predisposed to develop arthrofibrosis [6,9]. A study of patients with systemic sclerosis showed evidence of epigenetic changes in fibroblasts that may contribute to their continual activation [43]. In addition, microenvironmental changes, such as oxidative stress and hypoxia, play a pivotal role in mediating fibroblast proliferation, metaplasia and disease progression in tissues of patients with systemic sclerosis or those with Dupuytren's contracture, a fibrotic disorder of the hand [24,39,44]. Based on the observed increases in LDH activity and an anaerobic isozyme ratio (anaerobic LDH 4-5 versus aerobic LDH 1-3 isozyme expression), Ratajczak et al. (2007) concluded that hypoxia was involved in the pathophysiology of this disorder as well [44]. Interestingly, these two fibrotic disorders (systemic scerosis and Dupuytren's contracture) are also known to progress from tissue fibrosis to fibrocartilage formation and tissue calcification.

To determine the mechanism involved in the metaplastic progression of arthrofibrosis, we evaluated tissue hypoxia. We observed a decrease in tissue vascularization and an increase in LDH5 expression. Expression of LDH5 was restricted to the fibrotic and fibrocartilage regions of the arthrofibrotic tissue. Thus, the *in vivo *presence of LDH5 in fibroblasts and fibrocartilage confirms the previous *in vitro *findings, and suggests that therapeutic intervention to increase tissue vascularization may inhibit the progression of this disease process. Some patients do show a temporary improvement after physical manipulation of affected joints, which may increase blood flow to the region. However, manipulation alone is not sufficient to stop disease progression. As an alternative approach, low-dose irradiation has been shown to increase the release of vascular endothelial growth factor by activation of mast cells in an ischemic animal model, promoting vascular regeneration [45]. In the present study, we found increased numbers of mast cells in fibrotic regions of the arthrofibrotic tissue, suggesting that low-dose irradiation may be effective in treating these patients.

Mast cell proliferation and activation and increased expression of chymase have also been linked to fibroproliferative diseases, such as systemic sclerosis, a fibrotic disorder of connective tissue, and neurogenic bladder fibrosis [32,33,35]. The presence of chymase, a proteolytic enzyme, and the cleavage and activation of TGF-β have been associated with the development of scleroderma or systemic sclerosis of the skin [33,35]. The presence of increased mast cell numbers has also been observed after tissue injury in patients with fibrodysplasia ossificans progressiva, a disease marked by heterotopic ossification [38]. All three of these fibrotic diseases undergo metaplastic changes that progress to tissue calcification, suggesting an association between chymase-expressing mast cells and tissue metaplasia. The role of RONS in mediating mast cell degranulation is associated with both inflammatory cell and hypoxia-associated production of RONS [13,31]. Moreover, mast cell degranulation stimulates the hypoxia-associated increase in RONS, indicating that mast cells play a key role in hypoxic responses.

## Conclusion

In summary, our data implicates oxidative stress, chymase-expressing mast cells and hypoxia as driving forces for fibrosis and the metaplastic changes observed. Based on our findings, we suggest that tissue metaplasia in arthrofibrosis results from increased inflammatory-associated oxidative stress, leading to an accumulation of mast cells (secreting FGF), driving fibroblast proliferation and creating avascular regions of hypoxia. Hypoxia and associated oxidative stress induces the metaplastic conversion of fibrotic tissue to fibrocartilage, and subsequent bone formation by endochondral ossification, with larger bone fragments indicating a longer duration of fibrosis. Thus, hypoxia and associated oxidative stress present potential therapeutic targets for fibrosis and metaplastic progression of idiopathic arthrofibrosis after TKA.

## Methods

The study was performed in accordance with the institutional review board guidelines at Rush University Medical Center and The Rothman Institute of Orthopaedics at Thomas Jefferson University, and patients signed a consent form containing an agreement to volunteer surgical specimens for research.

### Tissue collection

This multi-center study used a standardized tissue retrieval protocol, allowing collection and analysis of periarticular knee tissues from patients undergoing revision arthroplasty for severe pain and stiffness. Diagnosis of idiopathic arthrofibrosis is based on clinical, radiological examination and intraoperative findings [8]. For these patients, the distinct intraoperative findings are extensive scar tissue formation that fills the lateral, medial and parapatellar gutters. All of the patients had undergone previous non-surgical attempts to alleviate pain and stiffness, including physical therapy. In all cases, other causes, such as infection or misalignment were ruled out as a cause of stiffness. Tissue samples from 10 affected knees of patients with arthrofibrosis and from 10 patients with osteoarthritis (OA) who were undergoing primary TKA were retrieved. Based on earlier arthrofibrotic studies by Ries *et al*., knees that were revised with only pain without stiffness, or with stiffness for other reasons, were not included in the arthrofibrotic group [6]. Radiographs from all patients were evaluated for heterotopic ossification. Primary surgical tissues were chosen for comparison with arthrofibrotic tissues, as OA is a chronic wound environment associated with inflammation and diffuse fibrosis regions that do not become extensively fibrotic or undergo metaplastic changes [46].

Tissue samples were taken from the periarticular area, which included the suprapatellar, medial gutter, lateral gutter and infrapatellar regions. The tissue was wrapped in gauze soaked in sterile saline, and transferred or shipped overnight on ice to the laboratory for fixation and detailed analyses. Tissue from each anatomical location was cut into pieces 2 × 5 mm in size and, depending on the amount of available tissue, five pieces of tissue from one region were placed in a paraffin wax block. An equal number and distribution of tissue pieces was used for fibroblast isolation. Any remaining tissue was flash-frozen in liquid nitrogen and stored at -80C.

### MicroCT analysis

Each of the paraffin blocks containing tissue were subjected to microCT analysis (μCT 40; Scanco Basserdorf, Switzerland) to determine heterotopic ossification, with an energy of 45 kVp, current of 88 μA and a 200-ms integration time, producing a resolution of 20 μm^3 ^voxel size. Each scan comprised a minimum of 500 slices through the entire paraffin block. To achieve image noise reduction, a constrained three-dimensional Gaussian filter (sigma 1.2, support 2) was applied. A fixed global threshold for analysis was chosen, which represented the transition in X-ray attenuation between unmineralized tissue (< 225 mg hydroxyapatite/cm^2^) and the forming bone (230-700). Analysis consisted of defining the outer boundary of the tissue for each 20 μm section in the sample. For consistency, the same settings and thresholds were used for each analysis, and applied to every sample in the study. Scout, sagittal and cross-sectional views were examined for evidence of mineralization.

### Histochemical stains

Tissues were fixed in 4% paraformaldehyde, dehydrated, embedded in paraffin and sectioned (6 μm). The paraffin blocks were stored at -20°C, and kept on ice between sections to allow for sectioning through regions containing small bone pieces. Paraffin sections were dewaxed, rehydrated and stained with Harris hematoxylin (catalogue number 245-678; Thermo Fisher Scientific Inc. Waltham, MA USA) and eosin Y (245-827; Thermo Fisher Scientific Inc.) to determine cellularity, vascularization and tissue morphology. Tissue calcification was determined with alizarin red S (130-22-3; Acros Organics, Morris Plains, NJ, USA). To assess proteoglycan content, the sections were stained with alcian blue 8GX (vA3157 Sigma Chemical Co., St Louis, MO, USA), and nuclei were identified with nuclear-fast red (EMD Chemicals;v6409-77-4). Tissue calcification was determined with alizarin red S as before. Slides were then mounted (Permount; Thermo Fisher Scientific Inc.) coverslipped, and evaluated by microscopy.

### TUNEL assay

Apoptosis was measured by the TUNEL assay, which takes advantage of the fact that during apoptosis, nuclear endonucleases digest genomic DNA into fragments of multiples of approximately 200 bp. To measure the fragmented DNA, the nucleotide ends were labeled using a commercial kit (*In Situ *Cell Death Detection Kit, Peroxidase diaminobenzidine [POD], 11 684 817 910; Roche Diagnostics, Indianapolis, IN, USA) according to the manufacturer's instructions, and fluorescence was visualized by microscopy as described below. Nuclei were stained with 10 μg/ml DAPI (D1306; Invitrogen, Carlsbad, CA, USA) in phosphate-buffered saline. DNase I recombinant, grade I (04 536 282 001; Roche Diagnostic) was used according to the manufacturer's instructions to generate TUNEL-positive control sections.

### Immunohistochemistry

Paraffin sections were cut at 6 μm and mounted on slides (Superfrost/Plus; Thermo Fisher Scientific Inc), which were placed in an oven at 58°C for 30 min before immunostaining. An automated slide stainer (Benchmark XT; N750-BMKXT-FS; Ventana Research, Pleasantan, CA, USA) was used for the immunohistochemical staining reactions. CC2 antigen (in citrate buffer, pH 6.0) retrieval was carried out for 36 min. Monoclonal anti-chymase antibody (CalBiochem, San Diego, CA, USA) was used at 1:200 dilution overnight at 4°C. Rabbit anti-nitrotyrosine (gift of Harry Ischiropoulos, University of Pennsylvania, PA, USA) was used at 1:1000 dilution for 1 hr at 37°C. LDH5 sheep polyclonal antibody (Ab9002-1; Abcam Inc. Cambridge, MA, USA) and Bcl-2 (C-2; Santa Cruz Biotechnology, Santa Cruz, CA, USA) were used at 1:50 dilution for 76 min at room temperature. Basic fibroblast growth factor (bFGF) antibody (Upstate, Lake Placid, NY) was used at 1:100 dilution overnight at 4°C. Secondary rabbit biotinylated anti-sheep IgG or anti-mouse (Vector Laboratories Inc., Burlingame, CA, USA) were used at 1:100 dilution. Antibodies were diluted in antibody diluent (DakoCytomation; Dako, Glostrup, Denmark). A slide stainer equipped with a diaminobenzidine (DAB) detection kit (iView; Ventana) was used for immunoperoxidase visualization of the targeted antigen. Endogenous biotin reactivity in the tissue sections was blocked (Endogenous Biotin Blocking Kit; Ventana). After completion of the staining run, the slides were briefly washed in a mild dishwashing detergent solution (Dawn; The Procter & Gamble Company, Cincinatti, OH, USA) to remove the liquid coverslip solution, and processed for hematoxylin counterstaining using a 1:8 dilution of Gills-3 hematoxylin solution (Polysciences, Warrington, PA, USA) for 1 min. Slides were then mounted (Permount; Thermo Fisher Scientific Inc.) coverslipped, and evaluated by microscopy. Secondary anti-mouse fluorescent dye (Alexa fluor 448; Invitrogen) was used at 1:200 dilution, and coverslipped using anti-fade mounting medium (Vectashield; Vector Laboratories Inc.) with DAPI.

### Image acquisition, capture and analysis

For each patient, 2-3 blocks of tissue were sectioned, and complete images of each section (25-30 individual images) were acquired at 20× magnification Images were acquired with a digital-cooled charge-coupled device (CCD) camera with RGB (red, green, blue) electronic filter (Retiga EXi QImaging, Burnaby, BC, Canada) or with an alternative digital camera (RT Color Spot; Diagnostic Instruments, Sterling Heights, MI, USA) using a microscope (either Optiphot or E800; Nikon, Melville, NY, USA). Image quantification was then performed (Image Pro Plus software; (Media Cybernetics, Silver Spring, MD, USA), using a customized macro to count DAB-stained cells and nuclei of cells stained with hematoxylin. A quantitative value of the inflammatory response was then presented as the average percentage of positive cells (DAB) per total cell number (hematoxylin) normalized to total area. The section results for each block from each anatomical site were averaged, and site differences compared.

### Statistical analysis

Differences between groups were analyzed using one-factor analysis of variance, and were considered significant at *P *< 0.05. All data passed normality and equal variance tests, and were analyzed using Student *t*-test with SPSS software (version-Base 13.0; SPSS Inc., Chicago, IL, USA).

## Competing interests

The authors declare that they have no competing interests.

## Authors' contributions

TAF was involved in the study design, analysis and data interpretation, and manuscript preparation. JP and CD contributed to the study design, data interpretation, and manuscript preparation. MJS was the study coordinator, and was involved in the study design, analysis and data interpretation and manuscript preparation. All authors read and approved the final manuscript.
